# A new species of *Chilicola* from Bahia, Brazil (Hymenoptera, Colletidae), with a key to the species of the *megalostigma* group

**DOI:** 10.3897/zookeys.153.2188

**Published:** 2011-12-09

**Authors:** Favízia Freitas de Oliveira, Thiago Mahlmann, Michael S. Engel

**Affiliations:** 1 Laboratório de Bionomia, Biogeografia e Sistemática de Insetos (BIOSIS), Departamento de Zoologia, Instituto de Biologia, Universidade Federal da Bahia, Rua Barão de Jeremoabo, s/n, Campus Universitário de Ondina, Salvador, CEP 40170-115, Bahia, Brazil; 2 Division of Entomology, Natural History Museum, and Department of Ecology & Evolutionary Biology, 1501 Crestline Drive – Suite 140, University of Kansas, Lawrence, Kansas 66045, USA

**Keywords:** Apoidea, Anthophila, Colletidae, Xeromelissinae, taxonomy, new species, *Chilicola*, *Hylaeosoma*, Brazil

## Abstract

The bee genus *Chilicola* Spinola (Xeromelissinae) is recorded from the State of Bahia, Brazil for the first time, based on a new species of the *megalostigma* group of the subgenus *Hylaeosoma* Ashmead. *Chilicola (Hylaeosoma) kevani* **sp. n.** is described and figured from males collected in Wesceslau Guimarães, Bahia. The species can be distinguished on the basis of coloration, size, integumental sculpturing, and structure of the hidden metasomal sterna and genitalia. A revised key to the species of the *megalostigma* group is provided.

## Introduction

The Neotropical genus *Chilicola* Spinola (Xeromelissinae) comprises small (ca. 3–8 mm in length), slender bees. The body is typically very long and usually black, without dense pubescence, and superficially resembling bees of the genus *Hylaeus* Fabricius (Hylaeinae). The genus occurs from Mexico to Chile, with its greatest diversity in Chile (Michener 1992, 1995, 2002, 2007; Packer and Genaro 2007). There are nearly 100 described species assigned to *Chilicola* and these are segregated into 15 subgenera (Packer 2008). While these subgenera appear to be good monophyletic units, the relationships among them are not entirely stable (Packer 2008).

The subgenus *Hylaeosoma* Ashmead has been thoroughly characterized by Michener (1992, 1995, 2002, 2007) and, unlike other subgenera, is not found in the temperate regions of South America, but occurs widely from Peru to northern Mexico (*e.g*., Michener 2002, 2007). Hitherto the subgenus has included 16 living and two fossil species (Table 1: Moure and Urban 2008). Michener (1992) divided the subgenus into two distinct groups of species, with those of the *megalostigma* group consisting of bees with polished and very shiny integument, elongate heads, and a flared and prominent preoccipital carina. Five species have been included in the group: *Chilicola (Hylaeosoma) megalostigma* (Ducke), *Chilicola (Hylaeosoma) polita* Michener, *Chilicola (Hylaeosoma) stenocephala* Brooks and Michener, *Chilicola (Hylaeosoma) yanezae* Hinojosa-Díaz and Michener, and *Chilicola (Hylaeosoma) muruimuinane* Smith-Pardo and Gonzalez. Four of the five species occur from Colombia to Mexico (Table 1), while *Chilicola megalostigma* is known from Peru, Bolivia, and Brazil (Moure and Urban 2008).

The present paper describes a sixth species of the *megalostigma* group, which also represents the first record of the genus *Chilicola* for the State of Bahia in Brazil. In addition, we provide an expanded and updated identification key to species for the *megalostigma* group.

**Table 1.** Species of *Chilicola* subgenus *Hylaeosoma*.

**Taxon**	**Sex known**	**Distribution**
Subgenus *Hylaeosoma* Ashmead		
*megalostigma* species group (= Group B, Michener 1992)		
*Chilicola megalostigma* (Ducke, 1908)	♀♂	Bolivia, Brazil, Peru
*Chilicola polita* Michener, 1992 [1994]	♀♂	Mexico to Costa Rica
*Chilicola stenocephala* Brooks & Michener, 1999	♀♂	Colombia: Amazonas
*Chilicola yanezae* Hinojosa-Díaz & Michener, 2005	♀♂	Mexico: Morelos
*Chilicola muruimuinane* Smith-Pardo & Gonzalez, 2007	♀♂	Colombia: Caquetá, Putumayo
*Chilicola kevani* **sp. n.**	♂	Brazil: Bahia
*longiceps* species group (= Group A, Michener 1992)		
*Chilicola longiceps* (Ashmead, 1900)	♀♂	Mexico: Jalisco; St. Vincent
*Chilicola huberi* (Ducke, 1908)	♀	Brazil: Ceará
*Chilicola aequatoriensis* Benoist, 1942	♀♂	Colombia, Ecuador, Peru, Venezuela
*Chilicola mexicana* Toro & Michener, 1975	♀♂	Mexico: México, Hidalgo, Morelos
*Chilicola griswoldi* Michener, 1992 [1994]	♀♂	Mexico: México, Michoacán
*Chilicola gracilis* Michener & Poinar, 1996	♂	Dominican amber (Miocene)
*Chilicola electrodominica* Engel, 1999	♀	Dominican amber (Miocene)
*Chilicola belli* Michener, 2002	♀♂	Colombia, Venezuela
*Chilicola canei* Michener, 2002	♀♂	Colombia: Antioquia
*Chilicola involuta* Michener, 2002	♀♂	Ecuador: Azuay
*Chilicola smithpardoi* Michener, 2002	♀♂	Colombia: Antioquia
*Chilicola umbonata* Michener, 2002	♂	Colombia: Valle; Ecuador: Loja
*Chilicola bochica* Gonzalez *in* Gonzalez and Giraldo 2009	♀♂	Colombia: Boyacá

## Material and methods

Morphological terminology used herein is adapted from Engel (2001) and Michener (2007), while the format for the description is taken from those of Hinojosa-Díaz and Michener (2005) and Smith-Pardo and Gonzalez (2007). Abbreviations used for common morphological terms are: S, metasomal sternum; T, metasomal tergum; F, flagellomere; DS, diameter of the antennal scape; and OD, ocellar diameter (based on the median ocellus). Measurements and proportions are adapted from Moure and Sakagami (1962): body length, head length and width, upper and lower ocular distances, and ocellocipital distance. Photomicrographs were prepared using a Nikon D1x digital camera attached to an Infinity K-2 long-distance microscope lens.

## Systematics

**Genus *Chilicola* Spinola**

**Subgenus *Hylaeosoma* Ashmead**

### Chilicola (Hylaeosoma) kevani

Oliveira, Mahlmann & Engel sp. n.

urn:lsid:zoobank.org:act:E90007EC-B88D-40D2-ABDD-CD5E5BB34D30

http://species-id.net/wiki/Chilicola_kevani

Figs 1 8

#### Holotype.

♂, Brazil, Bahia (Wesceslau Guimarães, Estação Ecológica, 18.I.2011 [18 January 2011], Rede Entomológica, P. Ferreira *Leg.* // Colletidae: *Chilicola (Hylaeosoma)* sp. n. ?, Det. Oliveira & Mahlmann, 2011 // Coletada na flor: Cyperaceae: *Scleria arundinacea* Kunth. The specimen is in excellent condition and is deposited in the Entomological Collection of the Zoological Museum of the Federal University of Bahia (MZUFBA), in Salvador, Bahia, Brazil.

#### Paratype.

♂, with same label data as holotype. Paratype deposited in the Division of Entomology (Snow Entomological Collections), University of Kansas Natural History Museum (SEMC), Lawrence, Kansas, USA.

#### Diagnosis.

**Figures 1–2.**
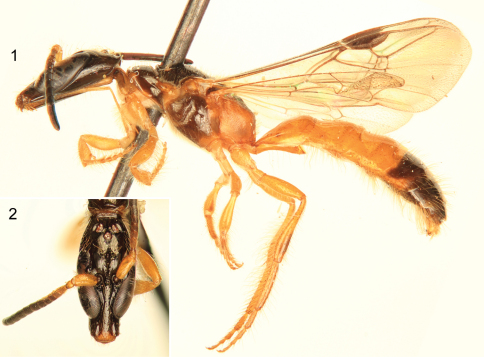
Photomicrographs of paratype (SEMC) male of *Chilicola (Hylaeosoma) kevani* Oliveira, Mahlmann, and Engel sp. n. **1** Lateral habitus **2** Facial aspect.

**Figures 3–8.**
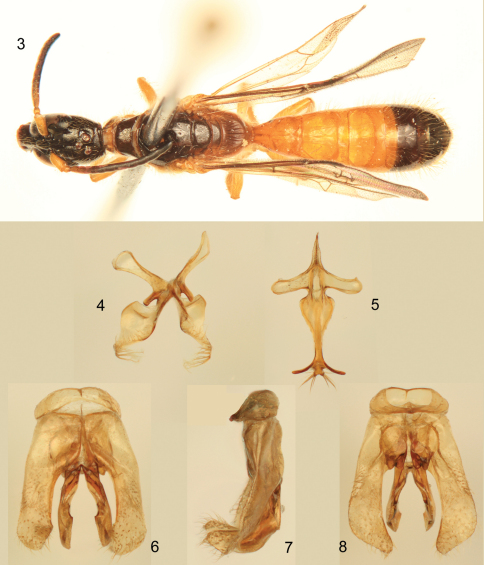
Photomicrographs of male paratype (SEMC) of *Chilicola (Hylaeosoma) kevani* Oliveira, Mahlmann, and Engel sp. n. **3** Dorsal habitus **4** Seventh metasomal sternum **5** Eighth metasomal sternum **6** Genital capsule, dorsal view **7** Genital capsule, lateral view **8** Genital capsule, ventral view.

This species is quite similar to other species of its group but differs from them in the markedly larger body size (ca. 7.6 mm), the largely honey yellow integument (Figs 1, 3) (except dark brown on head, flagellum, disc and sides of pronotum, mesoscutum, mesoscutellum, metanotal disc, basal dorsal surface of propodeum, most of the mesepisternum, and large portions of T4–T7 and S5–S6: refer to Description, *infra*), and the form of the hidden sterna (Figs 4, 5) and genital capsule (Figs 6–8).

#### Description.

♂: *Structure*: Total body length 7.60 mm; forewing length 4.70 mm. Head elongate (Fig. 2), length 2.12 mm, width 1.05 mm; compound eyes converging below (Fig. 2), upper ocular distance 0.65 mm, lower ocular distance 0.25 mm; paraocular depressed area well developed for reception of scape, terminating at level of upper tangent of compound eyes; area adjacent area to inner edge of depression above antennal alveoli with prominent gray oval spot, with diameter about 1OD and 2.5×longer than wide; F1 slightly longer than pedicel and about 2× longer than wide; F2 and F3 as long as broad; F11 approximately 3× longer than wide; malar area about 2× wider than long; occeloccipital distance about 2.5OD as measured from apex of preoccipital carina; preoccipital carina markedly laminate (Figs 1, 2); metasomal T1 longer than wide, length 0.90 mm, width 0.80 mm; T2 and T3 weakly constricted in basal half in profile; apex of T7 slightly truncate; distal margin of S6 terminating in two lateral lamellate projections from which arise a tuft of relatively long and thick bristles; S7, S8, and genitalia as in figures 4–8: S7 with two lateral projections between basal apodemes and more apical ventral and dorsal lobes, each lateral projection about 2× longer than wide (Fig. 4); apex of inferior lobe of S7 strongly narrowed and curved inward apically, outer edge with a comb of very long bristles (Fig. 4); superior lobe of S7 broad (Fig. 4); S8 basad of apodemes strongly narrowed, not forming a broad triangular area (Fig. 5); apical bifurcation of S8 nearly orthogonal to central axis of sternum, bearing small bilobed process between diverging processes, each lobe with prominent setae (Fig. 5); genital capsule as in figures 6–8, with gonostylus broadly rounded apically and curved ventrally near apex.

*Sculpturing*: Integument strongly polished and shining (as in other species of the *megalostigma* group), generally smooth or with very faint imbricate microsculpture, with scattered shallow punctures, most punctures separated by more than 2–3× a puncture width; two small, half-moon-shaped foveae on each side of head near concavity of compound eyes; dorsal base of propodeum with approximately 18 longitudinal carinae set in slight depression and radiating from basal margin.

*Coloration*:Integument predominantly honey yellow or amber colored (Figs 1, 3); head dark brown except labiomaxillary complex, labrum, apical margin of clypeus, scape, and pedicel honey yellow; F1 and F2 lighter brown than remainder of flagellum; pronotum largely brown with anterior margin and pronotal lobe honey yellow to amber colored; mesoscutum and mesoscutellum brown; metanotum and dorsal base of propodeum light brown; tegula translucent, honey yellow; axillary sclerites and base of C+Sc honey yellow, otherwise wing venation brown, pterostigma prominent and slightly darker brown than other veins; wing membrane hyaline, slightly and faintly infumate apically; majority of preëpisternum and mesepisternum brown; lower portion of metepisternum light brown; outer surface of metatibia slightly brownish, particularly in apical half; outer surface of metabasitarsus slightly brownish; distal margin of T3 with brown band interrupted medially (absent in paratype); T4–T7 brown except some honey yellow present at lateral extremities of T4; S5–S6 brown, with slightly brownish area apically on S4.

*Pubescence*:Mostly consisting of golden setae (Figs 1–3); head with scattered, largely simple setae, those on supraclypeal area, above compound eyes, vertex, gena, and postgena longer; setae dorso-apically on scape longer, ca. 1DS, remainder much shorter; a few short, branched setae on face near concavity of compound eyes. Mesosomal setae generally simple except more plumose around pronotal lobe; posterior margin of pronotum and lateral margins of mesoscutum with numerous, minute, pale, branched setae, becoming more whitish around pronotal lobe; discs of mesoscutum and mesoscutellum with relatively short and sparse setae; posterior margin of metanotum with minute whitish plumose setae interspersed with longer, gold setae, laterally with long setae, about 1.5DS, such setae apically curved; meso- and metapleura and lateral surface of propodeum generally with long, largely simple setae scattered over surface, although setae more numerous than on mesosomal dorsum, setae of preëpisternum slightly shorter and distinctly branched; pro- and metacoxae, protrochanter, and ventral surface of profemur with dense, long branched setae, such setae about 0.5× length of scape, density of setae on posterior of metacoxa about one-half that of procoxa; setae longer on inner surfaces of metatibia and metabasitarsus. Metasoma generally with sparsely scattered, long setae, mostly apically on terga and sterna, setae becoming progressively longer on more apical segments; lateral areas of S5 with very long, thick setae, extending to apex of metasoma (Fig. 1), such setae typically curved apically; S6 with lateral setae about one-third shorter, apically curved; setae distribution on S7–S8 and genitalia as in figures 4–8.

♀:Unknown.

#### Etymology.

The specific epithet is a patronym honoring Dr. Peter G. Kevan, University of Guelph, who has encouraged the study and highlighted the importance of pollinators in Brazil, particularly through field courses on pollination biology and ecology.

##### Key to species of the megalostigma species group

Modified and updated from keys provided by Brooks and Michener (1999) and Smith-Pardo and Gonzalez (2007).

**Table:** 

1	Body size small (4.5–6 mm); integument predominantly dark brown to black; male S7 with inferior apical processes broad, without prominent comb of strong bristles along margin; male S8 with broad triangular area surrounding spiculum, without constriction at base of elongate apical extension, apical bifurcating processes with acute angle between them, without small bilobed area between diverging processes	2
–	Body size relatively large (ca. 7.60 mm); integument predominantly honey yellow (Figs 1, 3); male S7 with inferior apical processes tapering rapidly to thin, elongate processes bearing prominent comb of strong bristles along margin (Fig. 4); male S8 basally narrowed around spiculum, strongly constricted at base of apical process, with diverging apical processes nearly orthogonal to longitudinal axis of sternum, with small bilobed area between diverging processes (Fig. 5) (Brazil: Bahia)	*Chilicola kevani* sp. n.
2(1)	Minimum distance between compound eyes about that of width of compound eye; malar area short, almost one-half or less than its maximum width; female basal metatarsomeres with apical process variable, ranging from almost straight to curved; male S8 more typical for subgenus and *megalostigma* group, elongate extension bifurcate apically, with thin diverging processes separated by an acute angle	3
–	Minimum distance between compound eyes about three-fourths width of compound eye; malar area long, more than one-half of its maximum width; female basal metatarsomeres with apical process distinctly curved; male S8 unique for subgenus, not bifurcate apically, apical extension spatulate, with broad apical area bearing prominent setae (Colombia: Amazonas)	*Chilicola stenocephala* Brooks & Michener
3(2)	Frontal line without depression; female basal metatarsomeres with apical process variable, with or without long thicker setae	4
–	Frontal line with conspicuous depression about 1OD in size just above level of antennal toruli, with antennal toruli forming a triangle of equal sides (isosceles); female basal metatarsomeres with apical process almost straight, terminating in a prominent setae thicker than other tarsal setae (Bolivia, Brazil, Peru)	*Chilicola megalostigma* (Ducke)
4(3)	Total length ca. 4.5–5.2 mm; pronotal coloration variable; male S7 with more elongate processes relatively broad	5
–	Total length ca. 5.5 mm; pronotum dark brown; female basal metatarsomeres with apical process almost straight, terminating in setae similar to other tarsal setae; male S7 with more elongate processes narrower, particularly in basal half (Mexico to Costa Rica)	*Chilicola polita* Michener
5(4)	Pronotum yellowish to light brown; female basal metatarsomeres with apical process almost straight, ending in a seta clearly thicker than other tarsal setae; male seventh sternum with conspicuous median projections bent ventrally, apicolateral process with subquadrate apex (Mexico: Morelos)	*Chilicola yanezae* Hinojosa-Díaz & Michener
–	Pronotum dark reddish brown; female basal metatarsomeres with apical process clearly curved, not terminating in a prominent seta; male seventh sternum without median projections, apicolateral process with broadly rounded apex (Colombia: Caquetá, Putumayo)	*Chilicola muruimuinane* Smith-Pardo & Gonzalez

## Discussion

Among the species of the *megalostigma* group perhaps one of the most unusual is *Chilicola stenocephala*. In this species the form of the terminalia differs dramatically from other members of the group and, indeed, from other *Hylaeosoma* as well. While species of the group tend to have S8 bifid apically, with the bifurcation comprising thin, diverging processing at the apex of a narrow elongate extension of the disc (e.g., Fig. 5), *Chilicola stenocephala* instead has a broad apical expansion bearing prominent setae [refer to figures in Brooks and Michener (1999)]. The gonostyli are also considerably different in this species in which they are narrowed apically, elongate, and curved mesally (Brooks and Michener 1999) in contrast to the otherwise broad and weakly or not curved mesally (typically curved ventrally) in the others species [e.g., Figs 6–8; and figures in Michener (1992, 2002), Hinojosa-Díaz and Michener (2005), Smith-Pardo and Gonzalez (2007)]. The remaining species have terminalia that are more or less of a similar structure. The S8 of *Chilicola megalostigma*, *Chilicola polita*, *Chilicola yanezae*, and *Chilicola muruimuinane* are the most similar in that each have a broadly triangular base encompassing the basal spiculum and extending to the lateral apodemes, while in *Chilicola kevani* this is greatly narrowed and the lateral apodemes are more prominent (e.g., Fig. 5). *Chilicola muruimuinane* perhaps comes closest in form to that of *Chilicola kevani* in that the sides of this triangular base are distinctly concave (Smith-Pardo and Gonzalez 2007), albeit not nearly as strongly so as in *Chilicola kevani*. In addition, while the aforementioned species have the discal process tapering rapidly to a narrow and elongate extension becoming bifid apically (a general structure somewhat characteristic of the *megalostigma* group), in *Chilicola kevani* there is a prominent constriction between the extreme base of the disc and the remainder of the apical portion of the sternum. Immediately apicad of the constriction the sternum flares outward, curves apicad and tapers rapidly to the narrow neck of the extension (Fig. 5). Apically the extension bifurcates with the thin processes diverging more strongly such that they are nearly orthogonal with the longitudinal axis of the sternum (Fig. 5). Between the processes is a small, apically bilobed structure which bears prominent setae at the apex of each lobe (Fig. 5), a unique autapomorphy among the subgenus. It is too early to comment on the possible interrelationships of these species as most features are autapomorphic and there are undoubtedly additional species to be discovered in the vast areas of suitable habitat throughout South America and southern Central America. Continued collecting in Bahia should be undertaken in order to discover the female of *Chilicola kevani*, to better document the distributions of the diversity of bee species in the region, and to document any new species that may come to light.

## Supplementary Material

XML Treatment for Chilicola (Hylaeosoma) kevani
